# Hemodynamics of speech production: An fNIRS investigation of children who stutter

**DOI:** 10.1038/s41598-017-04357-6

**Published:** 2017-06-22

**Authors:** B. Walsh, F. Tian, J. A. Tourville, M. A. Yücel, T. Kuczek, A. J. Bostian

**Affiliations:** 1 Department of Speech, Language, and Hearing Sciences, Purdue University, West Lafayette, IN USA; 2 Department of Bioengineering, University of Texas Arlington, Arlington, TX USA; 3 Department of Speech, Language, and Hearing Sciences, Boston University, Boston, MA USA; 4 Department of Radiology, Massachusetts General Hospital, Harvard Medical School, MGH/HST Athinoula A. Martinos Center for Biomedical Imaging, Charlestown, MA USA; 5 Department of Statistics, Purdue University, West Lafayette, IN USA; 6 Department of Speech, Language, and Hearing Sciences, Purdue University, West Lafayette, IN USA

## Abstract

Stuttering affects nearly 1% of the population worldwide and often has life-altering negative consequences, including poorer mental health and emotional well-being, and reduced educational and employment achievements. Over two decades of neuroimaging research reveals clear anatomical and physiological differences in the speech neural networks of adults who stutter. However, there have been few neurophysiological investigations of speech production in children who stutter. Using functional near-infrared spectroscopy (fNIRS), we examined hemodynamic responses over neural regions integral to fluent speech production including inferior frontal gyrus, premotor cortex, and superior temporal gyrus during a picture description task. Thirty-two children (16 stuttering and 16 controls) aged 7–11 years participated in the study. We found distinctly different speech-related hemodynamic responses in the group of children who stutter compared to the control group. Whereas controls showed significant activation over left dorsal inferior frontal gyrus and left premotor cortex, children who stutter exhibited deactivation over these left hemisphere regions. This investigation of neural activation during natural, connected speech production in children who stutter demonstrates that in childhood stuttering, atypical functional organization for speech production is present and suggests promise for the use of fNIRS during natural speech production in future research with typical and atypical child populations.

## Introduction

Although seemingly effortless, fluent speech production is a remarkably complex process, requiring the functional synergy of multiple neural networks to accomplish language formulation, articulatory planning, motor execution, and auditory and somatosensory integration^1–6^. Stuttering emerges in the preschool years as the brain develops the intricate and dynamically interactive networks underlying language formulation and speech production^7, 8^. Those who stutter know what they wish to communicate; however, involuntary disfluencies impede the forward flow of their speech. Examples of these include sound or syllable repetitions, blocks, and prolongations. Many factors, including heredity, linguistic abilities, speech motor abilities, and temperament, uniquely contribute to the onset of stuttering and in the likelihood of recovery or persistence^7–10^. Although most children who begin to stutter will recover, the approximately 25% who persist will struggle with a communication disorder that is resistant to treatment throughout their lives^11, 12^.

An extensive body of neuroimaging research in adults who stutter has provided greater insight into the neurological underpinnings of stuttering, confirming functional differences in speech and language network activation even when they are speaking fluently. Compared to fluent speakers, adults who stutter show heightened activity in right motor cortex and in cerebellar regions, but reduced activation in left anterior speech regions (e.g., premotor cortex-PMC, insula, inferior frontal gyrus-IFG, supplementary motor area-SMA)^13–21^. An additional finding in adults who stutter is anomalous degree and symmetry of activation in auditory cortices during speech production^13, 14^. Gray and white matter anomalies in perisylvian regions are presumed to induce atypical brain activity during speech production in adults who stutter^22–29^.

A growing number of anatomical imaging studies in children report decreases in gray matter volume in IFG and temporal regions bilaterally in children who stutter compared to controls, and reduced integrity of white matter tracts interconnecting auditory and motor speech areas^30–34^. However, there have been few functional neuroimaging investigations undertaken in children who stutter; thus, there are significant gaps in our understanding of speech neurophysiology closer to the onset of the disorder. The paucity of research in this population critically limits our ability to interpret findings from studies of adults who stutter and in turn, to inform developmental theories of stuttering. For example, it is unclear whether atypical lateralization of brain activity during speech production in adults who stutter represents a causal mechanism, or rather, a compensatory response, a supposition supported by studies of adults who stutter^14, 17, 18, 20, 35^. One magnetoencephalography (MEG) study found left lateralized activation of IFG preceding single-word production in both children who stutter and controls, suggesting that atypical speech lateralization in adults who stutter may not extrapolate to young children who stutter^36^. Clearly, more research into the neural correlates of speech planning and production in children who stutter is needed.

The neural regions implicated in stuttering are key components of a speech production network responsible for sequencing phonological codes to articulating sounds to communicate our message (for review^37^). Neural models of speech production posit that learned articulatory programs for commonly produced speech sounds are stored in left IFG and premotor cortex. Projections from these neurons to primary orofacial motor cortex encode the motor commands to articulate specific speech sounds. Bi-directional projections from the same neurons to superior temporal gyrus (STG) and lateral parietal cortex encode the expected sensory consequences of the articulatory movements. Any discrepancy between the expected and actual feedback is relayed back to motor cortex where it is used to correct and update the articulatory programs. This integration of sensory and motor information is key to the development and maintenance of fluent speech^1, 38–41^.

We have investigated the development of the neural control of speech production through our cross-sectional studies of lip and jaw speech kinematics in adults and children aged 4–16 years. We found that children’s articulatory patterns are highly variable and do not reach adult-like levels of proficient control and coordination until late adolescence^42, 43^. Moreover, we found that a significant number of young children who stutter are already showing a lag in their speech motor performance characterized by greater articulatory varibility^44^. These findings, at the behavioral level, provide another impetus to examine neurobiological mechanisms of speech motor development in typical children and in children with neurodevelopmental disorders such as stuttering.

Functional near-infrared spectroscopy (fNIRS) is a non-invasive, optical neuroimaging method that measures the relative concentration changes in oxygenated (Oxy-Hb) and deoxygenated (Deoxy-Hb) hemoglobin, two absorbing chromophores in cerebral capillary blood^45^. Like PET and fMRI, it offers an indirect index of neural activity. Stimulus-induced neuronal signaling increases metabolic demands resulting in measurable focal increases in regional cerebral blood flow(rCBF)^46^. The increased rCBF exceeds the oxygen metabolic rate of a region resulting in a peak in Oxy-Hb and a decrease or dip in Deoxy-Hb^47^. The Oxy- and Deoxy-Hb response from fNIRS measurements is spatially and temporally correlated to the blood oxygen level-dependent (BOLD) response from fMRI^48, 49^. fNIRS is a safe and more child-friendly neuroimaging approach^50, 51^ that allows silent, continuous recording of cortical hemodynamic responses during speech production^52–54^. fNIRS has been used to examine the regional activation, timing, and lateralization of brain activity for diverse perceptual, language, and cognitive functions (for review^55^). As such, fNIRS offers an unprecedented, practical means to examine the neurophysiology of speech production in typical and atypical child populations^56^.

As reviewed, there is strong evidence in both adults and children who stutter of atypical neuroanatomical development; however, we know comparatively little about the neurophysiology of sensory and motor regions underlying efficient timing and control of speech movements in children who stutter. At its core, stuttering manifests as a breakdown in speech production and is most likely to occur during connected, extemporaneous speech. Thus, it is critical to assess neural activation during more ecologically valid speaking conditions that place the greatest demands on the speech production networks of children who stutter.

The goal of this study is to examine hemodynamic responses from children who do and do not stutter over neural regions integral to fluent speech production and implicated in the pathophysiology of stuttering. These regions include the left and right IFG, the left and right precentral gyrus (premotor cortex-PMC), and the left and right STG. We hypothesize that connected speech, which maximally engages speech and language networks, will elicit a left-lateralized hemodynamic response over IFG for speech planning and production in the cohort of controls. There is evidence to support this from fMRI studies of picture description and narrative production in adult speakers^57, 58^ and from a recent fNIRS verbal fluency investigation with children and adults^59^. Based on earlier findings in stuttering adults (reviewed above), hemodynamic responses from children who stutter may show atypical symmetry and amplitude during speech production. We expect reduced responses, especially over left hemisphere IFG/PMC areas, and augmented responses over the right hemisphere homologous areas to compensate for left hemisphere neurophysiological deficits. Another possibility is that atypical neural activation patterns in adults who stutter result from years of compensating for their speech disorder, and thus, may not be present in children who stutter.

## Results

### Behavioral Data

One-way ANOVAs confirmed that there were no differences between the two participant groups in age *F*(1, 31) = 0.11, *p* = 0.74 and socioeconomic status (SES) *F*(1, 31) = 1.4, *p* = 0.24. Unusable trials (e.g., no responses, false starts, or data corrupted by motion) made up 4% and 3% of the total number of trials in the group of children who do and do not stutter, respectively. The between-group difference in the number of unusable trials was not significant (*F*(1, 31) = 0.09, *p* = 0.77. The two groups had a similar number of trials containing normal or typical speech disfluencies STUTT (*M* = 2.06; *SD* = 2.49) and CONT (*M* = 3.00; *SD* = 2.13) *F*(1, 31) = 1.31, *p* = 0.26. As expected, the children who stutter produced a greater number of stuttered trials compared to controls (STUTT *M* = 7.44; *SD* = 5.22; vs CONT *M* = 0.88; *SD* = 1.20). As a result, the group of children who stutter had significantly fewer fluent trials (*M* = 22.18; *SD* = 5.82) than the controls (*M* = 28.12; *SD* = 1.67) *F*(1, 31) = 19.53, *p* < 0.01. Finally, the two groups of children produced a comparable number of spoken syllables to describe each picture STUTT (*M* = 9.4; *SD* = 2.0) and CONT (*M* = 10.0; *SD* = 2.9), *F*(1, 31) = 0.41, *p* = 0.53. Examples of typical responses include, “A girl is watering the flower pots”. “There’s a tiger at the zoo with big teeth”. The correlation between age and number of syllables produced was significant (*r* = 0.61, *p* =  < 0.01). On average, the older participants produced longer responses to each picture.

### Hemodynamic responses

Table 1 provides the statistical summary from ANOVA. Separate ANOVAs on the amplitude of Oxy-Hb and Deoxy-Hb responses elicited during the null trials yielded no main effect of group, hemisphere or channel, or significant interactions between these factors. ANOVAs on the amplitude of Oxy-Hb responses for the talk (minus null) trials indicated no main effect of group, hemisphere, or channel; however, the channel X group and the hemisphere X channel X group interactions were significant. Similarly for Deoxy-Hb, there were no main effects; however, there was a significant channel X group interaction. To disentangle the interactions between group and within participant factors, separate independent sample *t*-tests were calculated to identify amplitude differences in Oxy-Hb and Deoxy-Hb responses from the group of children who stutter and controls for each channel. These results, along with means and standard errors for each channel are listed in Table 2.

**Table 1:** Statistical summary of group, hemisphere, and channel effects on hemodynamic responses for null and talk trials from ANOVA.

Main effect	Oxy-Hb			Deoxy-Hb		
	*F*-value	*p*-value	*df*	*F*-value	*p*-value	*df*
**Null Trials**						
Group	0.26	0.61	1	0.02	0.88	1
Hemisphere	0.37	0.55	1	1.7	0.20	1
Channel	0.98	0.41	3.06	0.37	0.74	2.54
**Interactions**						
Grp x Hem	0.34	0.86	1	0.01	0.99	1
Grp x Chan	0.51	0.68	3.06	0.36	0.75	2.54
Hem X Chan	0.68	0.58	3.19	1.20	0.31	2.79
Grp X Hem X Chan	2.06	0.12	3.19	1.47	0.23	2.79
**Talk Trials**						
Group	2.46	0.13	1	1.10	0.30	1
Hemisphere	0.06	0.81	1	0.06	0.82	1
Channel	0.57	0.69	3.97	0.51	0.72	3.80
**Interactions**						
Grp x Hem	1.98	0.17	1	1.28	0.27	1
Grp x Chan	2.87	0.03*	3.97	3.48	0.01*	3.80
Hem X Chan	0.29	0.88	3.84	0.82	0.50	3.61
Grp X Hem X Chan	4.64	<0.01*	3.84	2.70	0.04*	3.60

*p ≤ 0.05; *df*: degrees of freedom. Huynh-Feldt corrected *df* and *p*-values are reported when Mauchly’s Test of Sphericity was statistically significant.

**Table 2:** Relative Oxy-Hb and Deoxy-Hb concentrations *M* (SE) μM and independent-samples *t*-tests for between-group comparisons of each channel: children who stutter vs. controls.

Oxy-Hb							Deoxy-Hb					
Left Channels	STUTT	CONT	*t*-value	*p*-value	*df*		STUTT	CONT	*t*-value	*p*-value	*df*	
1 IFG	0.08 (0.06)	0.01 (0.08)	0.65	0.52	30		−0.01(0.03)	−0.03(0.03)	0.48	0.64	30	
2 IFG	0.02 (0.06)	0.12 (0.09)	−0.89	0.38	30		−0.04(0.02)	−0.04(0.04)	0.04	0.97	30	
3 IFG	0.02 (0.04)	0.18 (0.09)	−1.63	0.11	16.93		0.01(0.02)	−0.06(0.04)	1.64	0.12	20.22	
4 IFG	−0.11 (0.03)	0.17 (0.05)	−4.84	<0.01*	21.62	Stutt < Cont	0.04(0.01)	−0.06(0.03)	3.03	<0.01*	20.07	Stutt > Cont
5 IFG	−0.13 (0.06)	0.19 (0.05)	−4.21	<0.01*	30	Stutt < Cont	0.03(0.02)	−0.11(0.03)	3.75	<0.01*	30	Stutt > Cont
6 STG	0.11 (0.09)	−0.07 (0.08)	1.53	0.14	30		−0.07(0.04)	0.05(0.04)	−1.95	0.06	30	
7 STG	0.10 (0.08)	−0.01 (0.07)	1.10	0.28	30		−0.05(0.05)	0.03(0.04)	−1.16	0.26	30	
8 PMC	−0.08 (0.05)	0.18 (0.06)	−3.35	<0.01*	30	Stutt < Cont	0.01(0.03)	−0.11(0.03)	3.08	<0.01*	23	Stutt > Cont
9 PMC	−0.06 (0.06)	0.14 (0.04)	−3.85	<0.01*	30	Stutt < Cont	0.01(0.04)	−0.09(0.02)	2.20	0.04	23	
**Right Channels**												
1 IFG	0.05 (0.07)	−0.03 (0.08)	0.79	0.44	30		−0.01(0.02)	0.02(0.03)	−0.71	0.49	30	
2 IFG	0.06 (0.04)	0.11 (0.08)	−0.55	0.59	22.61		−0.04(0.03)	−0.04(0.04)	0.04	0.97	30	
3 IFG	0.07 (0.04)	0.10 (0.08)	−0.35	0.73	30		−0.03(0.02)	−0.05(0.03)	0.65	0.52	30	
4 IFG	0.04 (0.05)	0.06 (0.07)	−0.26	0.80	30		0.003(0.02)	−0.04(0.03)	1.07	0.29	30	
5 IFG	0.01 (0.05)	0.05 (0.06)	−0.63	0.53	30		−0.04(0.03)	−0.04(0.03)	−0.10	0.99	30	
6 STG	0.04 (0.07)	0.11 (0.05)	−0.79	0.43	30		−0.06(0.05)	−0.02(0.03)	−0.65	0.52	30	
7 STG	0.04 (0.05)	−0.01 (0.04)	−1.91	0.07	30		−0.01(0.04)	−0.04(0.03)	0.54	0.59	30	
8 PMC	−0.003 (0.08)	0.11 (0.06)	−1.19	0.11	30		0.02(0.04)	−0.05(0.03)	1.31	0.20	30	
9 PMC	−0.03 (0.04)	0.08 (0.04)	−2.01	0.05	30		0.01(0.03)	−0.05(0.04)	1.22	0.23	30	

*p ≤ 0.05, FDR-corrected. If Levene’s Test for Equality of Variances was statistically significant for a comparison, *t-*statistics and degrees of freedom were adjusted using the Welch-Satterthwaite method. *df* = degrees of freedom; IFG = inferior frontal gyrus, STG = superior temporal gyrus, PMC = premotor cortex.

Finally, the correlations between age or number of syllables and Oxy-Hb amplitudes for channels collapsed across hemisphere and region of interest in either group were not significant. The correlation between stuttering severity and average Oxy-Hb amplitudes in the group of children who stutter was also not significant (*r* = −0.30–0.38; all *p* > 0.05).

### Speech-Evoked Hemodynamic Responses over IFG

**Figure 1:**
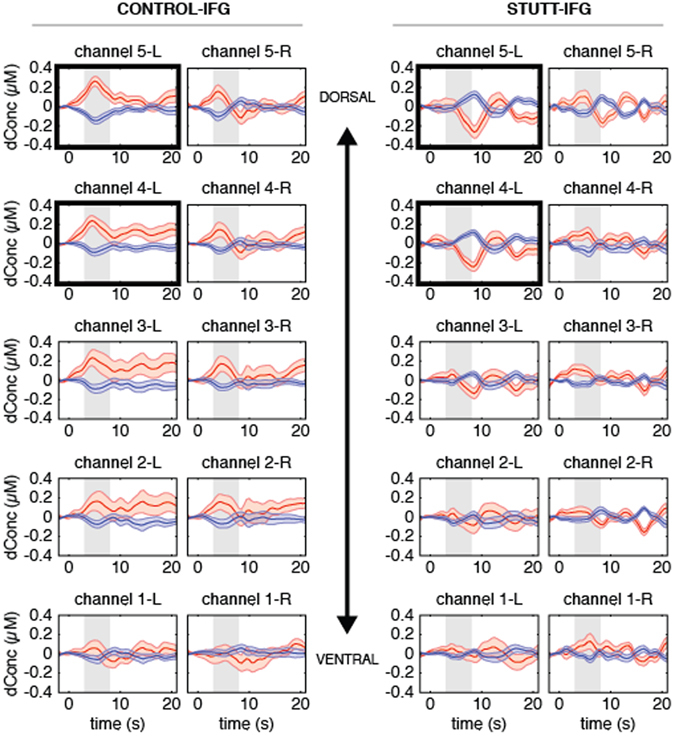
Grand averaged Oxy-Hb (red) and Deoxy-Hb (blue) hemodynamic responses with standard error curves for IFG channels. Channels for the controls (n = 16) are shown on the left hand side of this figure and channels for the children who stutter (n = 16) are plotted on the right. Within each participant group, left hemisphere channels are plotted side-by-side with homologous right channels for comparison. Channels showing a significant group difference are bold-framed. Time = 0 indicates trial onset. Gray shading indicates the 3–8 s. window of analysis.

To observe the overall responses from the control group, the temporal profiles of Oxy-Hb (red) and Deoxy-Hb (blue) hemodynamic responses were grand-averaged across the 16 children in this group and presented on the left side of Fig. 1. Overall, controls’ hemodynamic responses over IFG showed a canonical increase in Oxy-Hb, accompanied by a dip in Deoxy-Hb, characteristic of neural activation^60^. Oxy-Hb and Deoxy-Hb concentration amplitudes reached a peak approximately 5–6 seconds after stimuli onset. Higher amplitudes of Oxy-Hb responses, averaged within the 3–8 s post-stimulus window of analysis, were elicited from left dorsal IFG channels compared to left ventral IFG channels. We noted similar, albeit reduced, hemodynamic responses over right IFG (Fig. 1). The within group paired *t*-test confirmed higher Oxy-Hb amplitudes over left compared to right IFG (Table 3).

**Table 3:** Within group paired-sample *t-*tests for laterality comparisons of Oxy-Hb response amplitudes collapsed across channels for each ROI: left vs. right.

CONTROLS *df* = 15	Oxy-Hb		
	*t*-value	*p*-value	
IFG	2.29	0.04*	L > R
STG	−2.07	0.06	
PMC	1.48	0.16	
**CHILDREN WHO STUTTER** ***df*** = **15**			
IFG	−2.60	0.02*	R > L
STG	1.19	0.26	
PMC	−1.06	0.30	

*p ≤ 0.05. *df* = degrees of freedom; IFG = inferior frontal gyrus, STG = superior temporal gyrus, PMC = premotor cortex.

The grand-averaged waveforms on the right side of Fig. 1 reveal notably different hemodynamic responses in left IFG channels for the group of 16 children who stutter. In channels over left dorsal IFG, there was little change in Oxy-Hb and Deoxy-Hb responses early in the 3–8 s analysis window, a time when the control group showed clear increases in Oxy-Hb/dips in Deoxy-Hb in these same channels. This was followed by a distinct negative dip in Oxy-Hb accompanied by a peak in Deoxy-Hb around 8–9 s. We did not detect this atypical hemodynamic profile in right IFG channels. A pairwise *t*-test confirmed significantly reduced amplitudes of Oxy-Hb responses in left compared to right IFG for the group of children who stutter (Table 3).

### Speech-Evoked Hemodynamic Responses over STG and PMC

**Figure 2:**
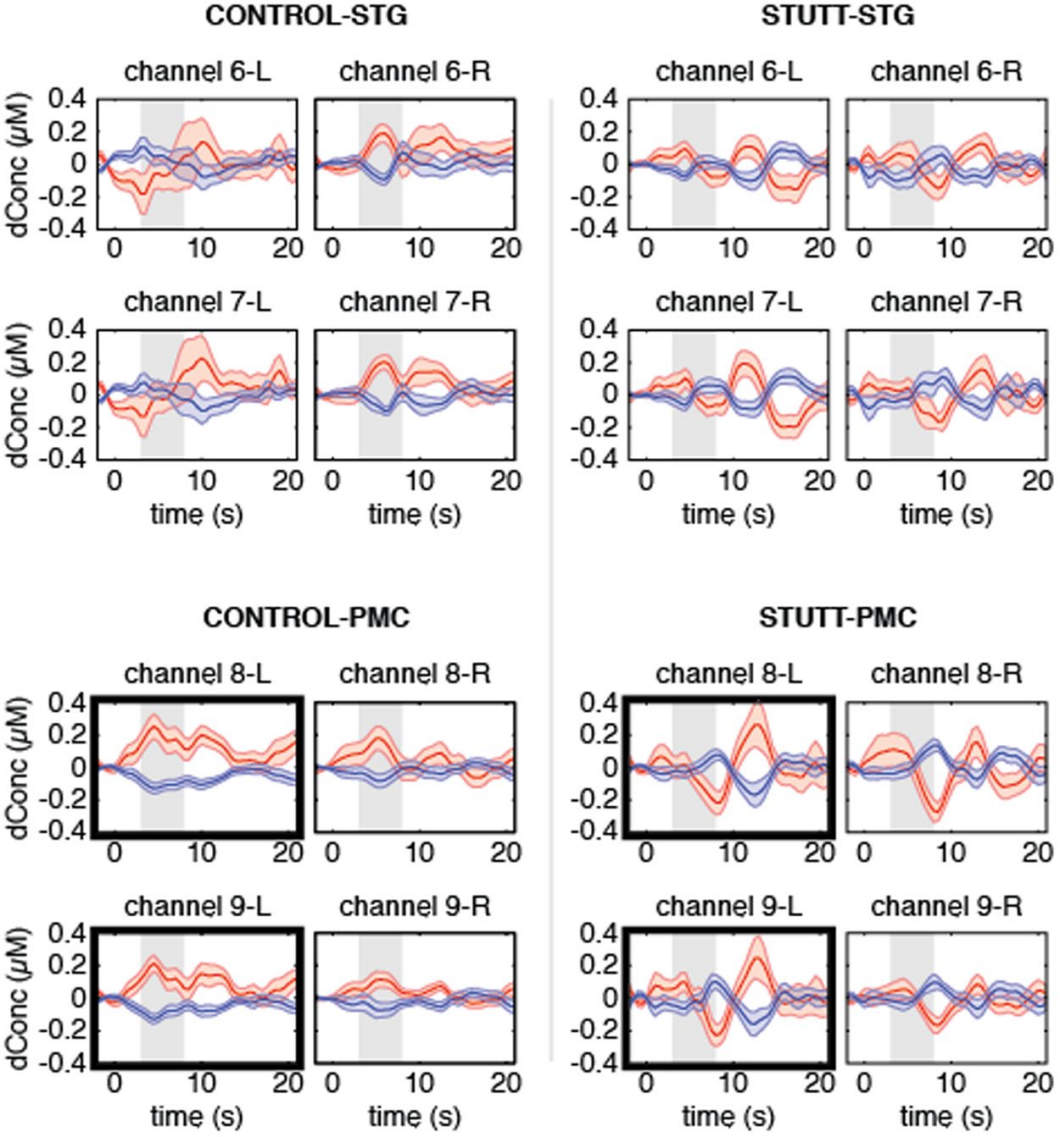
Grand averaged Oxy-Hb (red) and Deoxy-Hb (blue) hemodynamic responses with standard error curves for STG (top plots) and PMC (bottom plots) channels. Channels for the controls (n = 16) are shown on the left hand side of this figure and channels for the children who stutter (n = 16) are plotted on the right. Within each participant group, left hemisphere channels are plotted side-by-side with homologous right channels for comparison. Channels showing a significant group difference are bold-framed. Time = 0 indicates trial onset. Gray shading indicates the 3–8 s. window of analysis.

Figure 2 shows the grand-averaged Oxy-Hb and Deoxy-Hb hemodynamic responses for left and right STG and PMC with controls on the left side of this figure. In the group of controls, we saw an early dip in Oxy-Hb over left STG followed by a later positive peak approximately 8–10 s after stimuli onset. The average Oxy-Hb and Deoxy-Hb responses over left STG were variable, denoted by the error curves in this figure. There was a peak in Oxy-Hb over right STG approximately 6 s after stimulus onset. The amplitude of Oxy-Hb responses were higher in right STG compared to left STG, although this difference was not statistically significant (Table 3). Finally, Oxy-Hb and Deoxy-Hb responses over left and right PMC channels peaked approximately 5–6 s post-stimulus onset with a second peak occurring around 10 s in left PMC channels. The amplitudes of Oxy-Hb responses in left and right PMC were also not statistically different (Table 3).

The grand-averaged Oxy-Hb and Deoxy-Hb hemodynamic responses from STG and PMC channels for the group of 16 children who stutter are shown on the right hand side of Fig. 2. There was modest activation of left and right STG channels, and the amplitudes of Oxy-Hb responses in left and right STG were not statistically different (Table 3). The hemodynamic responses over PMC shared similar characteristics with left IFG channels characterized by little change in Oxy-Hb and Deoxy-Hb early in the window of analysis followed by negative peaks in Oxy-Hb/positive peaks in Deoxy-Hb approximately 8–9 s post-stimulus onset. The left vs right PMC comparison was also not significant for the group of children who stutter (Table 3).

### Comparison between Controls and Children Who Stutter

**Figure 3:**
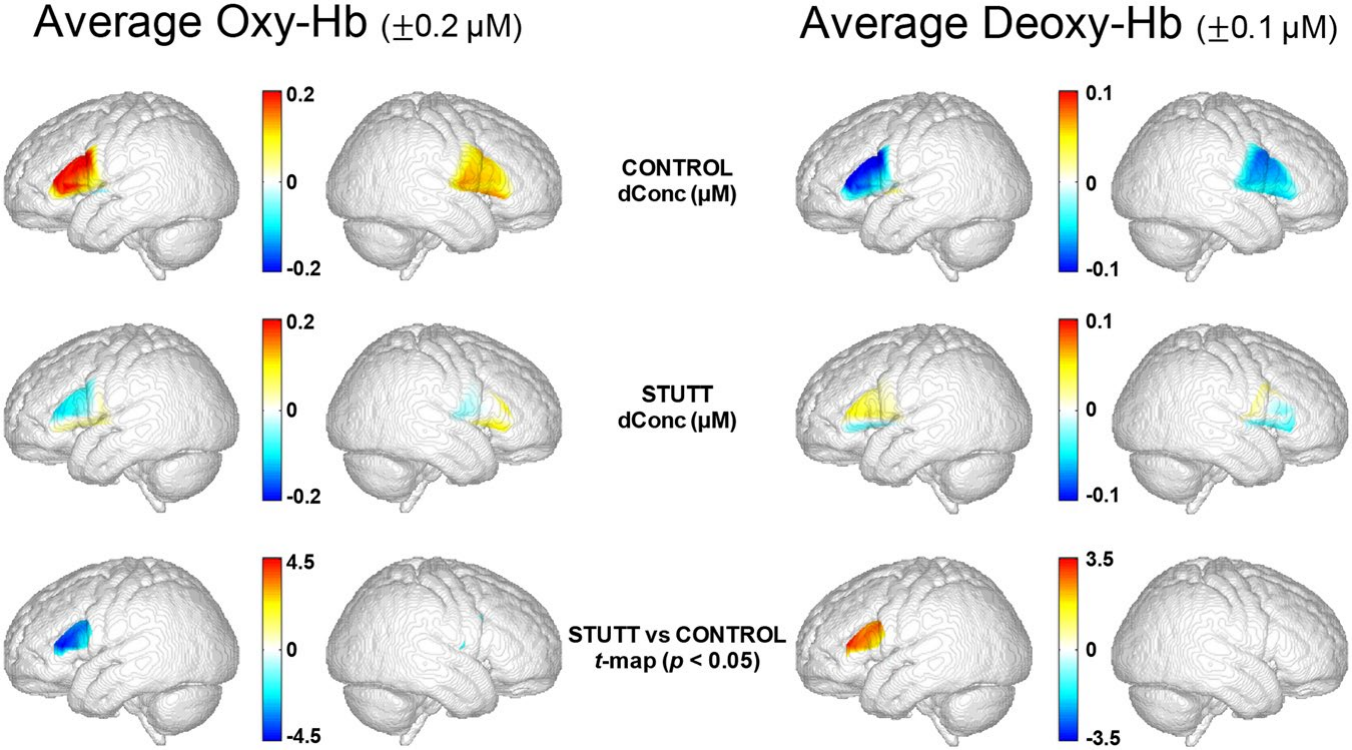
Topographic images of cortical activation within the boundaries of the probe derived from Oxy-Hb (left pairs) and Deoxy-Hb (right pairs) response amplitudes within the 3–8 s post-stimulus analysis window of analysis. First rows: left and right hemisphere average hemoglobin responses for the control participants. Second rows: left and right hemisphere average hemoglobin responses for group of children who stutter. Third row: *t*-maps outlining regions of significant difference between the control and stuttering groups (independent sample *t*-tests, *p* < 0.05, FDR-corrected). The brain templates, courtesy of John Richards’s Lab, represent average whole-head MRI images from 72 10-year-old children.

Independent sample *t*-tests confirmed the children who stutter had significantly reduced Oxy-Hb responses in left dorsal IFG (channels 4 and 5) and left PMC (channels 8 and 9) compared to the controls (Table 2; Figs 1 and 2). The group of children who stutter also had significantly larger Deoxy-Hb responses in left IFG (channels 4 and 5) and left PMC (channel 8). Figure 3 shows topographic images of speech-evoked average Oxy-Hb and Deoxy-Hb responses in the 3–8 s window within the boundaries of the probe. The differences in hemoglobin responses between children who stutter and controls were significant over the left hemisphere (Fig. 3, third row images). No between-group comparison reached statistical significance for any right hemisphere channel. In order to appreciate the range of values from individuals in each group, average Oxy-Hb response amplitudes for the talk trials are provided in Appendix A for the channels showing a significant between-group difference.

## Discussion

To our knowledge, this is the first investigation of brain activation during connected speech production in children who do and do not stutter. IFG, PMC, and STG are key components of an intricate speech network that develops atypically in adults and children who stutter; thus providing a clear rationale for targeting these neural regions. We employed a slow event-related picture-description task that allowed us to record cortical activation during overt speech production. Comparing the speech-evoked hemodynamic responses from each group, we found children who stutter had markedly different hemodynamic responses compared to their fluent peers. While controls exhibited hemodynamic profiles showing clear increases in Oxy-Hb and decreases in Deoxy-Hb over left hemisphere dorsal IFG and PMC, responses from the group of children who stutter showed deactivation characterized by decreases in Oxy-Hb (and increases in Deoxy-Hb) over these left hemisphere regions.

The differences in hemodynamic profiles that we detected may indicate deficient planning and production processes related to stuttering. Insufficient engagement of left hemisphere IFG and PMC is a reasonable explanation for the reduced or missing early peaks in Oxy-Hb in the stuttering group; however, it cannot explain the abrupt decreases in Oxy-Hb that immediately followed within the window of analysis. The interpretation of negative hemodynamic responses and whether they signify neuronal deactivation, or alternatively, a shift in blood flow to supply activation of adjacent neural regions provokes much debate^61, 62^. Although we may only speculate at this point, one explanation is that the deactivation is compensatory and indicates a shift in blood flow to regions outside of the probe boundaries to compensate for functional deficits in the primary areas. People who stutter report the ability to anticipate moments of stuttering^63^ and may use idiosyncratic coping mechanisms to avoid or mask their disfluencies. What the listener perceives as fluent speech may in fact be “covert stuttering” or other compensatory strategy requiring significant cognitive effort to circumvent overt breakdowns in speech production. The neurophysiological correlates of increased cognitive effort during speech production have not been measured; however, future fNIRS investigations with expanded probe coverage could assess whether greater blood flow is detected in areas adjacent to IFG/PMC, for example, to prefrontal areas that may mediate the conscious avoidance of speech breakdowns.

An alternative explanation for deactivation of left IFG/PMC in the stuttering group is a disruption of cortical-subcortical interactions resulting in a net inhibition of these regions. Dopamine hyperactivity in the basal ganglia has been associated with stuttering^64, 65^. Using the GODIVA/DIVA computational modeling framework to simulate production of multisyllabic speech production, Civier and colleagues demonstrated stuttering-like disfluencies (syllable onset delays) when dopamine levels were atypically high in model’s putamen analog^66^. The high dopamine levels also resulted in a clear net reduction in the activity in the component of the model that stores speech motor programs hypothetically located in left ventral premotor and adjacent posterior inferior frontal cortex. In the present study, the deactivation of left inferior frontal and premotor cortex in children who stutter occurred during fluent speech production. Thus, it may reflect atypical processes that underlie stuttering, for example, disrupted cortico-basal ganglia circuits as suggested above, that have been corrected or that fail to reach a threshold that results in a disfluency.

As reviewed, there is precedence for reduced activation and compromised functional and structural interconnectivity of speech circuitry in children and adults who stutter. Desai and colleagues noted significantly reduced resting state regional cerebral blood flow to Broca’s area in adults and children who stutter^67^. Deactivation of frontal and temporal speech regions was reported by an earlier PET study in adults who stutter, although this anomaly was reversed when fluency was induced through choral reading^16^. Finally, decreased frontal and temporoparietal activation for the planning and production of both speech and nonspeech stimuli in adults who stutter has been shown with fMRI^14^. According to a meta-analysis of PET and fMRI studies with adults who stutter, neural activation for fluent speech in adults who stutter is characterized by underactivity in left motor regions accompanied by a right hemisphere shift in activity in the stuttering group^68^. Although not specifically delineated, right hemisphere compensation for left hemisphere deficits has been proposed in some capacity to explain findings of reversed laterality of neural activation in adults who stutter^17, 20, 21^. We did not detect “overactivation” of right hemisphere channels in the children who stutter; right hemisphere responses did not significantly differ between the groups. Rather, our findings suggest that atypical left, rather than right IFG/PMC hemodynamic responses, motivated the significant differences between the two groups of children (Fig. 3).

Earlier functional imaging studies in adults who stutter relied upon less natural speaking tasks such as choral speech, word generation, oral reading, speaking in time with a metronome, or producing overlearned content. We recorded fluent and disfluent speech tokens during a more natural speaking task. The present study focused exclusively on fluent responses, allowing us to compare hemodynamic responses between groups of children who do or do not stutter (i.e, stuttering “trait”^68^). A follow-up study in children who stutter examining hemodynamic responses from both types of trials (i.e., fluent and disfluent) is underway that may help clarify current findings. For example, if hemodynamic response patterns that we found for fluent speech are wholly different from hemodynamic responses recorded during stuttered speech, then this may provide support for compensatory mechanisms.

Although not statistically significant, the trajectories of the hemodynamic responses for the two channels over left and right STG appeared dissimilar in the group of controls (Fig. 2), with the channels over left STG showing an early decrease in Oxy-Hb followed by a late positive peak in Oxy-Hb. The responses from left STG channels were highly variable; thus, it is unclear whether we detected a response specific to the left hemisphere, for example suppression to one’s own speech^69^, or if this was a spurious result. To address this, a probe providing denser coverage of STG is needed, a caveat we consider below.

Finally, we did not find significant correlations between Oxy-Hb response amplitude and stuttering severity (Stuttering Severity Index 3^rd^ Edition) within the group of children who stutter. We will examine whether the correlation between severity and Oxy-Hb amplitude for stuttered speech in a follow-up study with children who stutter. It may be the case, however, that average hemoglobin response amplitude is too coarse a metric to differentiate between a mild or moderate stuttering problem. A focus for future research, in our lab and others, is to explore the use of pattern recognition or feature selection algorithms to classify participants into their respective groups by comparing, for example, temporal features from an individual’s data with group averages^70^. Ichikawa and colleagues utilized responses from a subset of fNIRS channels to classify children with 84% accuracy into either attention-deficit/hyperactivity-ADHD or autism spectrum disorder-ASD groups^71^.

We acknowledge several limitations of the present study. First, we were careful to place the probe consistently and symmetrically over the same head locations on each of our participants using EEG 10–20 markings. We also obtained stereotaxic coordinates with a digitizing stylus and registered them to a child MRI template^72^. However, there could still be variation in placement due to experimenter error or individual anatomy. Although we believe that this did not affect the group average results or our main conclusions, replication of these findings is critical. Secondly, although IFG received denser coverage in terms of the number of channels spanning this region, two channels over STG may not adequately capture activation of a relatively large neural region associated with diverse cognitive, language, and auditory processes. Finally, we placed sources and detectors a sufficient distance apart in our probe arrays to minimize potential contributions from hemodynamic fluctuations in the scalp. However, we did not include separate indices of systemic physiology. In follow-up investigations, probe arrays could accommodate short separation channels, which will allow separation of systemic contributions from brain responses^73^. However, with both of these caveats in mind, securing the optodes in place to elicit reliable signals is time consuming, and with child populations, compromises must be made to optimize obtaining complete data sets from most participants.

The results from this study advance the potential of using fNIRS to examine neural activation during natural speech production and indicate its promise as an innovative research tool to study speech perception and production deficits associated with other speech and language disorders. Although the neural substrates of speech and language processes are well-defined in adults, a recent review of the fMRI literature from the past two decades reveals few investigations into the functional development of these networks in children^74^. The 15 studies of expressive language selected for this review all relied upon single word production (i.e., word generation or verbal fluency tasks). Moreover, 10 out of the 15 studies elicited covert responses. As this review clearly highlights, there is a need for more ecologically valid investigations of language development. Although fNIRS has coarser spatial resolution compared to fMRI, it is well suited to examine the activation of broad neural regions and to investigate the functional lateralization of brain activity^55^. This study represents a vital step toward taking this research into younger populations of children who stutter near onset to determine whether the aberrant hemodynamic responses in school-aged children are indicative of a higher risk for developing a persistent stuttering problem.

## Methods

### Participants

Thirty-two children participated in the study, 16 children who stutter (13 boys) and 16 SES and age-matched controls (11 boys). The participants were between the ages of 7 and 11 years (STUTT, *M* = 9.1 yrs, *SD* = 1.5 years); (CONT, *M* = 9.2 yrs, *SD* = 1.4 yrs). Per parent report, all children were native speakers of North American English with no history of cognitive, developmental, neurological, or speech and language disorders other than stuttering in the group of children who stutter. Children were screened for medications affecting the central nervous system (e.g., depressants, stimulants, analgesics, etc.). The participants scored within normal limits on the Clinical Evaluation of Language Fundamentals Screening Test (CELF-IV^75^)and passed a bilateral pure-tone hearing screening at 500, 1000, 2000, 4000, and 6000 Hz at 20 dB. All participants were right-handed, assessed with the Handedness Questionnaire^76^. Finally, the two groups had comparable SES determined by the parents’ level of education^77^. SES was evaluated on a 7-point scale (1 = *less than 7* ^*th*^ *grade education* to 7 = *completion of a graduate or professional degree*). Criteria for the diagnosis of stuttering and general characteristics of the children who stutter are provided in Appendix B and C, respectively.

### Task and Procedures

**Figure 4:**
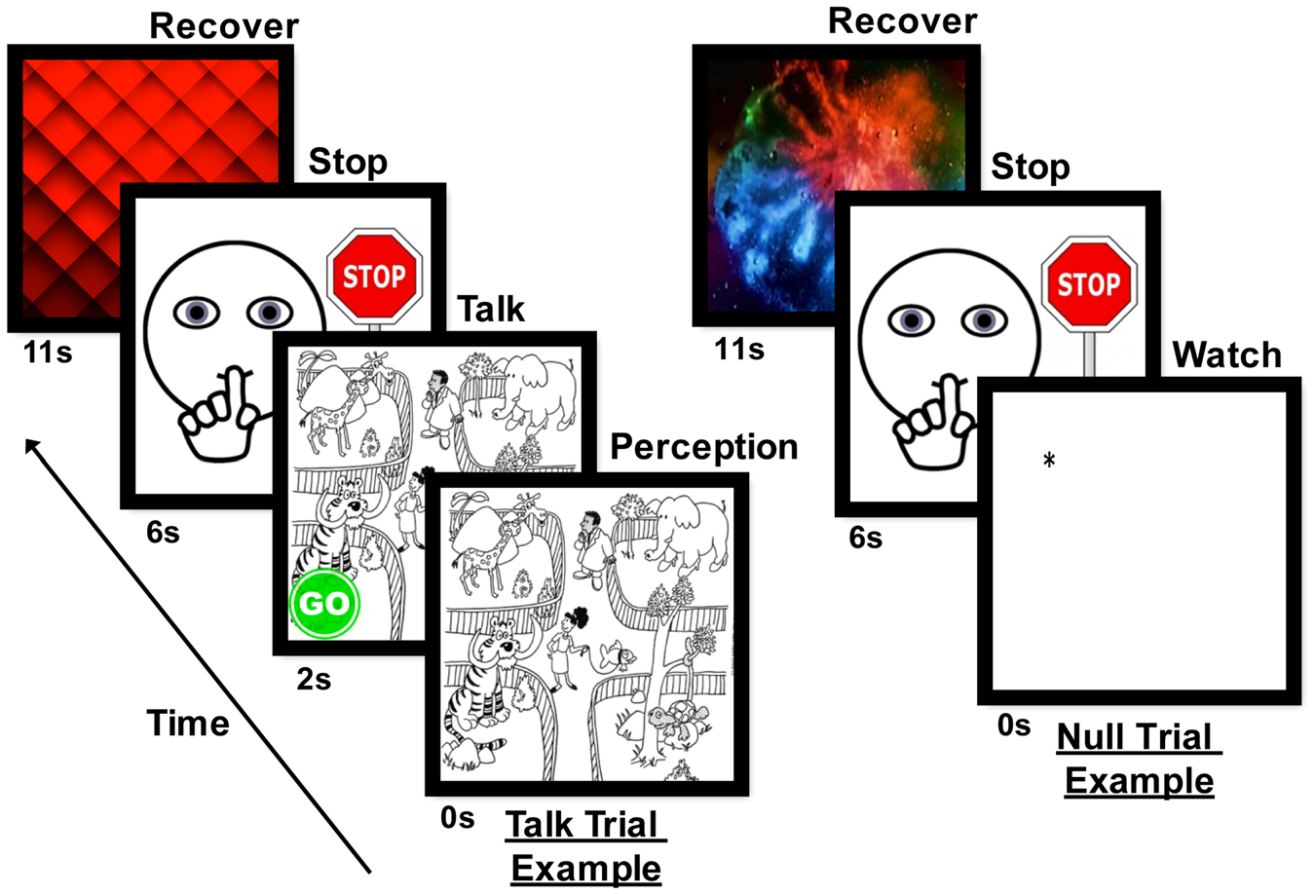
Experiment overview. Example of a talk trial (left slides) and a null trial (right slides). For the 30 talk trials, children viewed each picture for 2 s. A green “go” circle appeared in the corner of the picture cueing the children to begin speaking. After 4 s of speech, a stop sign appeared cueing the children to stop speaking. Finally, a patterned slide, devoid of overt semantic content, remained on the screen for 10–12 s to allow the hemodynamic response to recover. For the 15 null trials, the children watched a fixation point for 6 s (the amount of time they would have seen and described a picture) followed by the a stop sign and patterned recovery slide. The talk and null trials were randomized for presentation. Zoo illustration used with permission from Super Duper® Publications.

The research protocol was approved by the Institutional Review Board at Purdue University and adhered to Human Subjects research regulations and guidelines. After obtaining written informed consent from parent(s)/legal guardian and written informed assent from the children, the participants were seated approximately 50 in. in front of a 24 in. computer monitor. The chair height was adjusted so the monitor was at the child’s eye-level. As shown in Fig. 4, stimuli for the talk trials were 30 child-friendly black and white illustrations^78^. The picture stimuli were presented using E-prime software (2.0, Psychology Software Tools, Inc., Sharpsburg,PA) on a PC synchronized with the data acquisition unit to insert precise onset markers into the continuous fNIRS recording at the beginning of each trial. We employed a slow event-related design in which each trial began with an activation interval to allow the hemodynamic response to reach its maximum followed by a rest interval for recovery^79^. The null trials were included to reduce habituation to the talk trials, and critically, to establish a baseline condition in which no language formulation and speech production occurred to contrast with the talk trials in subsequent analyses. This approach, used in earlier language-related studies with children, allowed us to target speech-related activation evoked by the picture description task (e.g. refs 80–82). Before data collection began, the participants completed training to ensure they understood and could perform the task accurately. During the training period, the children were told that they would, “First look at, and then tell us what is happening in each picture”. After training, the children understood that they were to describe the scenes using full sentences, and not to simply list the items they saw. The experiment was video and audio recorded and lasted approximately 18 minutes including a provided break in the middle.

### Functional Near-Infrared Spectroscopy Recording

#### Instrumentation

**Figure 5:**
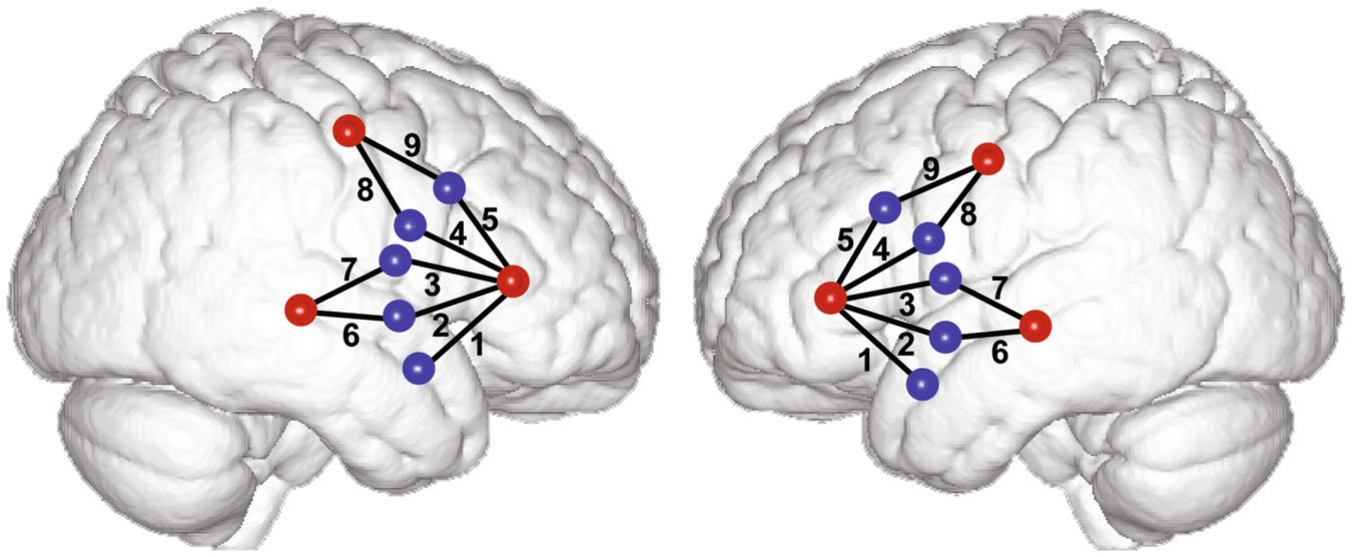
fNIRS probe arrays. Approximate positions of emitting (red circles) and detecting (blue circles) optodes are shown on a standard brain atlas (ICBM 152). The probes were placed symmetrically over both hemispheres, with channels 1–5 spanning inferior frontal gyrus, channels 6–7 over superior temporal gyrus, and channels 8–9 over precentral gyrus/premotor cortex.

The participants’ cerebral hemodynamic changes were recorded during the experiment with a continuous-wave fNIRS system (TechEn, Inc., Milford, MA). The system uses near-infrared lasers at 690 and 830 nm as light sources, and avalanche photodiodes (APDs) as detectors for measuring intensity changes in the diffused light at a 25-Hz sampling rate. To ensure reliable placement of the optodes over regions of interest, two customized probes (one for each hemisphere) were created. First, we designed a template of the left and right hemisphere layout based upon International 10–20 system coordinates (e.g. refs 83, 84). As shown in Fig. 5, each of the left and right probes contained three sources and five detectors. The distance between each source-detector pair, henceforth referred to as a channel, was 2.8 cm with targeted brain regions lying approximately at the midpoint of a channel. The left and right probe each contained 9 channels: channels 1–5 recorded over ventral and dorsal IFG including BA 44/45/47^85–87^, channels 6–7 recorded over STG^88, 89^, and channels 8–9 over precentral gyrus/PMC^90, 91^. For consistent probe placement, we made markings on each participant’s scalp denoting left and right probe boundaries using customized 10–20 EEG caps (EasyCap, Herrsching, Germany). After the EEG caps were removed, the probes were placed symmetrically over each hemisphere utilizing these markings^83, 92^.

#### Spatial Registration

After the probe was secured on each participant, his/her five fiducial points (i.e., nasion, inion, left and right preauricular points, and vertex) and the location of each optode were recorded with a digitizing stylus (Polhemus; Colchester, VT). In order to estimate the cortical regions that the fNIRS probe covered, a spatial registration procedure^93^ was performed on 15 participants (11 males, 4 females) representing the age range of the participants^94, 95^. This procedure converted the real-world stereotaxic coordinates of the optodes from the digitizing stylus to stereotaxic coordinates in a 10-year-old brain template. The template (downloaded with permission from: http://jerlab.psych.sc.edu/NeurodevelopmentalMRIDatabase/index.html) was generated as an average of whole-head images from 72 children (10; 0–10; 4 years) scanned by 1.5 T or 3.0 T MRI^72^. Age-specific MRI brain templates created from typically developing participants provided an approximation of head and brain structures of participants in this study.

### Data Processing and Analysis

#### Behavioral Data

Speech output was recorded with a TASCAM linear PCM digital recorder and video (with accompanying audio) was captured with a Toshiba HD Camcorder fitted with a preamplifier. The participants’ utterances were transcribed by one experimenter online during the experiment, and confirmed with video and audio during offline analysis. A syllable count was obtained for each picture.

#### fNIRS Data

The fNIRS data was preprocessed using Homer2 software^96^. Only channels showing clear, real-time cardiac pulses were used in subsequent analyses. Data corrupted by large movement artifact (e.g., yawns, coughs) appearing as large shifts from baseline were manually removed from the record. Trials in which the participant failed to respond, responded before the go sign, continued speaking after the stop sign, or produced less than three syllables were not included in subsequent analyses. The remaining trials were classified as null, talk (i.e. fluent speech), or stutter (disfluent speech). The aim of the present study is to compare hemodynamic responses elicited from children who do and do not stutter. Therefore, only fluent speech trials were considered in the analyses.

The raw data were band-pass filtered (0.03–0.5 Hz) to remove slow drift and high frequency noise and converted into changes in optical density. Next, the concentration changes in Oxy-Hb and Deoxy-Hb were calculated based on the Modified Beer-Lambert Law which accounts for scattering properties of tissue with a differential pathlength factor of 6.0^97^. In order to correct for speech-related motion artifacts due to jaw movement, we employed a correlation-based signal improvement (CBSI) approach^98^. CBSI is a channel-by-channel subtraction procedure applied to Oxy-Hb and Deoxy-Hb data, and is a valid method for reduction of speech-related motion artifacts^99^.

After motion artifact correction, we derived each participant’s averaged Oxy-Hb and Deoxy-Hb hemodynamic response from each channel from 0 s (stimulus onset) to 21 s post-stimulus onset across trials of the same condition (i.e., talk or null). We used the 2 seconds prior to stimulus onset to set the baseline of each hemodynamic response, which is a standard option in Homer2. In order to specify neural activation exclusively associated with speech production, we subtracted the averaged hemodynamic response from a child’s null trials from the averaged talk response to derive a “differential” hemodynamic response for each channel^100^. Next, the average concentrations of Oxy-Hb and Deoxy-Hb of these responses were calculated within a 3–8 s post-stimulus onset window for each channel. This window was specifically targeted to accommodate individual differences in task-evoked activation peaks while considering the temporal lag of the hemodynamic response^101^.

### Topography

Topographic images were generated using a Matlab-based optical topography toolbox, EasyTopo^94^, based on the 10-year-old brain template^72^ and corresponding stereotaxic coordinates obtained from the spatial registration procedure. This toolbox implemented 2D angular interpolation to generate topographic images of brain activation by approximating the cortex as a hemispherical surface. In this study, the channel-wise amplitudes of Oxy-Hb and Deoxy-Hb responses within the 3–8 s window were used to generate activation maps for the talk condition for each group, and the channel-wise *t*-statistic values reflecting the statistical power of difference between the two groups were used to generate the *t*-maps for group comparison.

### Statistical Analysis

One-way analyses of variance (ANOVAs) were calculated to ensure that the groups were matched for age and SES and to compare behavioral performance during the experimental task. To assess the amplitudes of Oxy-Hb and Deoxy-Hb responses during the null trials and the talk trials, we computed separate repeated-measures ANOVAs with group (controls and children who stutter) as a between-subjects factor, and hemisphere (left and right), and channel (9) as within-subject factors. In cases where the assumption of sphericity was violated, Huynh-Feldt corrected degrees of freedom and *p*-values are reported. If interactions between group and within-subject factors reached significance, independent sample *t*-tests (two-tailed) were calculated employing the false discovery rate (FDR) correction to correct for multiple comparisons (*p* < 0.05)^102^. To examine the laterality of activation, we calculated paired-samples *t*-tests comparing the amplitudes of Oxy-Hb responses for left and right regions of interest (collapsed across channels) for each group. Finally, we calculated Pearson bivariate correlation analyses to examine potential relationships between amplitudes of Oxy-Hb responses from channels collapsed across hemisphere and region of interest and behavioral variables (i.e., age, number of syllables produced, and stuttering severity).

## Electronic supplementary material

Appendices
