# Supplementary material for: Hemodynamics of speech production: An fNIRS investigation of children who stutter
Source: Sci Rep. 2017 Jun 22;7:4034. doi: 10.1038/s41598-017-04357-6 (PMC5481456; doi:10.1038/s41598-017-04357-6)
Supplement: Supplementary file 1 — Appendices [file 41598_2017_4357_MOESM1_ESM.pdf]

Supplementary Material

Article Title: Hemodynamics of speech production: An fNIRS investigation of children who stutter

Authors: [1]Walsh, B., [2]Tian, F., [3]Tourville, J. A., [4]Yucel, M. A., [5]Kuczek, T., [6]Bostian, A. J.

Author Affiliations:
[1] *Bridget Walsh, Ph.D., CCC-SLP
Purdue University, Department of Speech, Language, and Hearing Sciences
West Lafayette, IN

[2] Fenghua Tian, Ph.D.
University of Texas Arlington, Department of Bioengineering
Arlington, TX

[3] Jason A. Tourville, Ph.D.
Boston University, Department of Speech, Language, and Hearing Sciences
Boston, MA

[4] Meryem A. Yücel, Ph.D.
Massachusetts General Hospital, Harvard Medical School, MGH/HST Athinoula A. Martinos Center for Biomedical Imaging, Department of Radiology
Charlestown, MA

[5] Thomas Kuczek, Ph.D.
Purdue University, Department of Statistics
West Lafayette, IN

[6] Anna J. Bostian
Purdue University, Department of Speech, Language, and Hearing Sciences
West Lafayette, IN

Corresponding Author:
***Bridget Walsh, Ph.D., CCC-SLP**
Purdue University
Department of Speech, Language, and Hearing Sciences
Lyles Porter Hall
715 Clinic Dr.
West Lafayette, IN 47907-2122
(765) 496-0151
(765) 494-0771 (fax)
bridget@purdue.edu

Appendix A. Individual data points with overlying box plot of average Oxy-Hb concentration for channels showing a significant between-group difference.

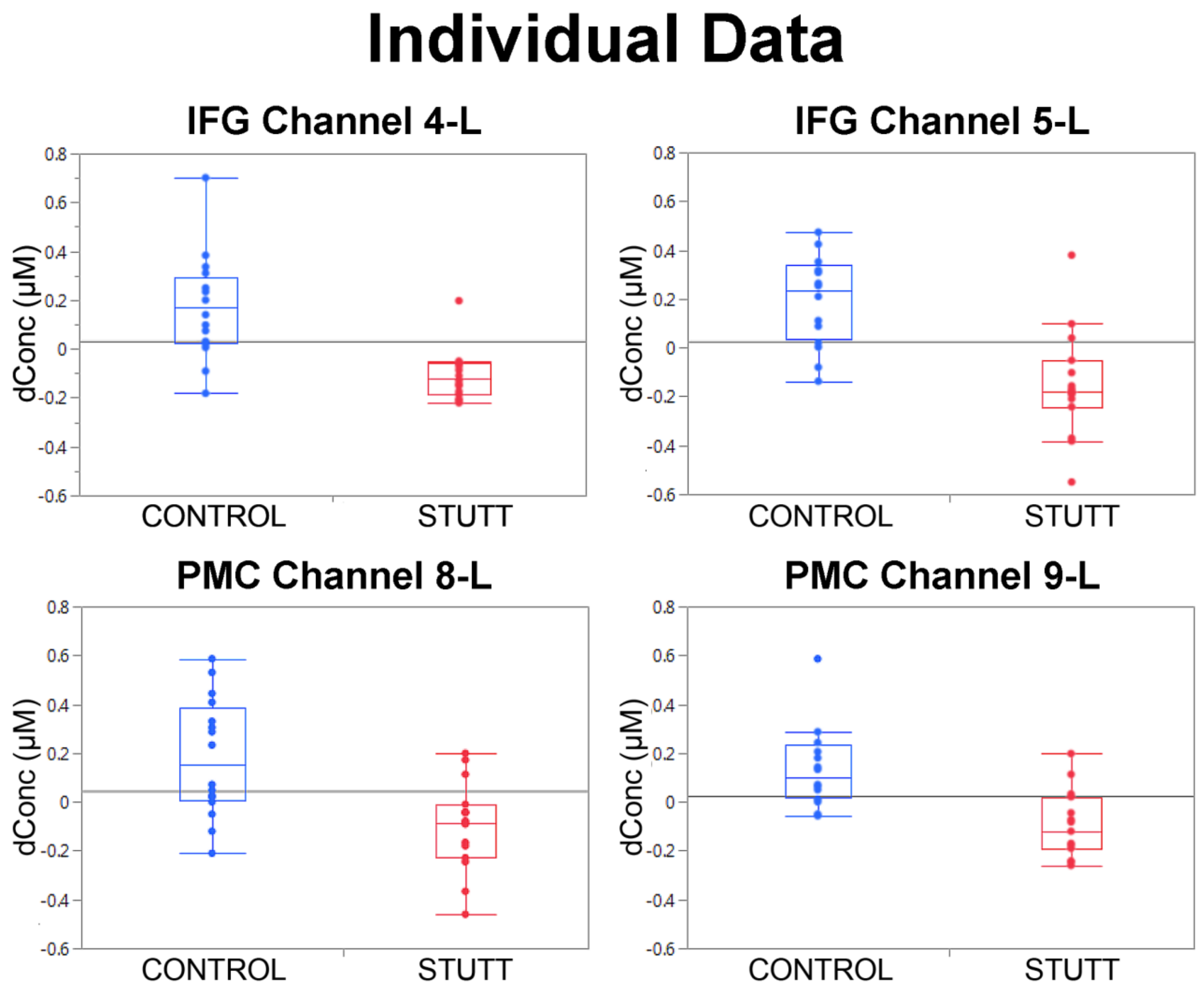

Appendix B. Criteria for the diagnosis of stuttering

(1) evidence of at least 3% stuttered syllables during conversational and reading tasks, (2) score at least "very mild" on the Stuttering Severity Instrument Third Edition (SSI-3; Riley, 1994), and (3) be considered stuttering by both the child's parent(s)/legal guardian and by a speech-language pathologist (first author). Children who stutter ranged in severity from very mild to moderate on the SSI-3. The average duration of stuttering (i.e., time since onset) was 6 years (*SD* = 2 years) according to parent report. Finally, children who stutter were eligible to participate in the study regardless of whether they had received, or were currently receiving speech therapy. We documented approximately 69% of the children who stutter (11/16) had received, or were currently receiving speech therapy. Characteristics of the children who stutter are provided in Appendix C.

Riley, G. D. *Stuttering severity instrument for children and adults.* (Pro Ed, 1994).

Appendix C. General characteristics of the children who stutter (CWS)

**Participant**	**Sex**	**Age** (yrs; months)	**Time Since Onset** (yrs)	**History of Speech Therapy**	**SES**	**SSI Score**	**SSI Severity Index**
CWS 1	M	8;1	5	N	7	9	very mild
CWS 2	F	9;5	7	Y	6	23	moderate
CWS 3	M	8:0	6	Y	4	18	mild-mod
CWS 4	M	10;0	7	Y	6	19	mild-mod
CWS 5	M	11;6	7	N	7	9	very mild
CWS 6	M	11;2	N/A	Y	6	18	mild-mod
CWS 7	M	9;0	6	N	7	23	moderate
CWS 8	M	8;3	4	N	5	18	mild-mod
CWS 9	F	11;1	8	Y	6	11	mild
CWS 10	M	11;0	9	Y	7	7	very mild
CWS 11	M	9;0	6	Y	6	21	moderate
CWS 12	M	7;9	5	Y	6	13	mild
CWS 13	M	7;0	4	Y	6	13	mild
CWS 14	M	8;9	4	Y	7	24	moderate
CWS 15	M	8;1	5	Y	6	12	mild
CWS 16	F	7;1	4	N	7	9	very mild
